# Evaluating the Value of Defensins for Diagnosing Secondary Bacterial Infections in Influenza-Infected Patients

**DOI:** 10.3389/fmicb.2018.02762

**Published:** 2018-11-20

**Authors:** Siyu Zhou, Xianwen Ren, Jian Yang, Qi Jin

**Affiliations:** ^1^MOH Key Laboratory of Systems Biology of Pathogens, Peking Union Medical College, Institute of Pathogen Biology, Chinese Academy of Medical Sciences, Beijing, China; ^2^BIOPIC, School of Life Sciences, Peking University, Beijing, China

**Keywords:** viral infection, bacterial infection, diagnosis, defensin, gene expression

## Abstract

Acute respiratory infections by influenza viruses are commonly causes of severe pneumonia, which can further deteriorate if secondary bacterial infections occur. Although the viral and bacterial agents are quite diverse, defensins, a set of antimicrobial peptides expressed by the host, may provide promising biomarkers that would greatly improve the diagnosis and treatment. We examined the correlations between the gene expression levels of defensins and the viral and bacterial loads in the blood on a longitudinal, precision-medical study of a severe pneumonia patient infected by influenza A H7N9 virus. We found that DEFA5 is positively correlated to the blood load of influenza A H7N9 virus (*r* = 0.735, *p* < 0.05, Spearman correlation). DEFB116 and DEFB127 are positively and DEFB108B and DEFB114 are negatively correlated to the bacterial load. Then the diagnostic potential of defensins to discriminate bacterial and viral infections was evaluated on an independent dataset with 61 bacterial pneumonia patients and 39 viral pneumonia patients infected by influenza A viruses and reached 93% accuracy. Expression levels of defensins in the blood may be of important diagnostic values in clinic to indicate viral and bacterial infections.

## Introduction

Acute respiratory infections by influenza viruses are commonly the causes of severe pneumonia, which can further deteriorate if secondary bacterial infections occur (McCullers, 2014). Accurate detection of influenza virus infections and the potential secondary bacterial infections is important to improve the diagnosis and treatment of patients with severe pneumonia. Because the viral and bacterial agents are quite diverse, seeking a broad-spectrum test based on only the characteristics of pathogens is currently still a challenging task. Although the rapidly developed next-generation sequencing (NGS) technology provides a powerful tool to catalog the taxonomic composition of clinical samples, the great technological complexity and high price makes it hard to adopt in clinic soon. Identifying biomarkers that can be readily adopted into clinic is urgently needed. Because different pathogens can result in convergent host responses, identifying broad-spectrum diagnostic biomarkers from the host response is probable. With the rapid development of high-throughput biomedical technologies, the gene expression profiles of host blood can now be readily obtained. Recently several groups have reported in succession that the gene expression profiles of a certain set of genes in the human blood can robustly discriminate bacterial infections from viral infections and a series of bioinformatics tools have been developed to identify the associations between microbes and host health (Ramilo et al., 2007; Edelman et al., 2009; Zaas et al., 2009; Parnell et al., 2012; Hu et al., 2013; Mejias et al., 2013; Peng et al., 2013; Ye et al., 2014; Suarez et al., 2015; Sweeney et al., 2016; Tsalik et al., 2016; Huang Y. A. et al., 2017; Huang Z. A. et al., 2017; Wang et al., 2017; Chen et al., 2018), suggesting the great potential of host response as the diagnostic signature.

Defensins are diverse members of a large family of antimicrobial peptides that are considered as an important part of the innate immune response of hosts and are found in many compartments of the body (Ganz, 2003). These great properties of defensins indicate that they may be good candidates of diagnostic biomarkers to discriminate bacterial/viral infections. However, the currently reported gene signatures identified with human blood gene expression profiles seldom include defensins. It is of pressing need to find out the clinically diagnostic values of defensins.

To reach the objective, we profiled the gene expression levels in blood and the viral and bacterial loads in plasma of a severe pneumonia patient infected by influenza A H7N9 virus via the next-generation sequencing (NGS) technology along with the disease progression. Then we examined the correlations between the expression levels of defensins and the viral and bacterial loads in the blood. Although many defensins did not demonstrate statistically significant correlations with either the viral or the bacterial loads, the *p*-values of several defensins did reach the statistical significance cutoff after multiple-testing corrections. And these statistically significant defensins demonstrated mutually exclusive correlations with the viral loads and the bacterial loads, suggesting that defensins are of great diagnostic values to discriminate viral and bacterial infections. Upon this observation, we then examined the diagnostic potential of defensins on an independent dataset with 61 bacterial pneumonia patients and 39 viral pneumonia patients infected by influenza A viruses (Parnell et al., 2012) via a machine learning method, which confirmed again that defensins are of great diagnostic values to discriminate bacterial infections from viral infections. These results suggest that expression levels of defensins in the blood may be of important diagnostic values in clinic to indicate viral and bacterial infections.

## Materials and methods

### Longitudinal gene expression profiles of a severe pneumonia patient infected by influenza a H7N9 virus

The severe pneumonia patient infected by influenza A H7N9 virus was admitted to hospital on Day 5 after illness onset and died on Day 29. Since Day 6, blood samples were collected for every three days, i.e., on Days 6, 9, 12, 15, 18, 21, 24, and 27 after illness onset. The total RNA was isolated and then subjected to sequencing on Illumina Solexa GA II with read length of 80 bp (see Hu et al., 2015 for the technical details). Cufflinks (version 2.1.1, with default parameters) (Trapnell et al., 2010) was used to quantify the gene expression profiles of defensins after mapping the quality-controlled reads to human genome (GRCh37 and Gencode19) using Tophat (version 2.0.10, with default parameters) (Kim et al., 2013). This study was reviewed and approved by the Ethics Committee of the Institute of Pathogen Biology, Chinese Academy of Medical Sciences and Peking Union Medical College. Written informed consent was obtained for the use of peripheral blood samples from the patient's relatives. This study was carried out in accordance with the recommendations of the Institute of Pathogen Biology, Chinese Academy of Medical Sciences and Peking Union Medical College. The protocol was approved by the Institute of Pathogen Biology, Chinese Academy of Medical Sciences and Peking Union Medical College. All subjects gave written informed consent in accordance with the Declaration of Helsinki.

### Quantifying the microbial species infecting in the blood samples

To quantify the microbial species infecting in the blood samples, a metagenomic analysis method was applied. In detail, the same sequencing reads were aligned to the NCBI non-redundant nucleotide database by BLASTN (version 2.2.22, with parameters “-e 1e-10 –b 10 –v 10”) (Altschul et al., 1997). Then, the results were parsed and visualized by the MEGAN software (Huson et al., 2007, 2016; Mitra et al., 2011), upon which those reads specifically mapped to bacterial or viral genomes were counted and exported as the bacterial/viral loads in each sample. To facilitate comparisons among samples, the bacterial/viral loads were normalized by sequencing depth (i.e., the total sequencing reads obtained for each sample).

### Evaluating correlations of defensin levels and bacterial/viral loads

Spearman's rank correlation coefficient (Spearman, 1987) was then used to evaluate the associations between defensins and viral/bacterial loads. Specifically, given the expression levels of a defensin at all the eight time points *x*_*i*_ where *i* = 1, …, 8 and the normalized loads of a specific bacterial/viral species *y*_*j*_ where*j* = 1, …, 8, ranks *r*^*x*^ and *r*^*y*^ were firstly obtained and then the correlation was calculated according to the following formula:

(1) $$\begin{array}{r} r_{xy}=\frac{\mathrm{cov}\left(r^{x},r^{y}\right)}{\sigma_{r^{x}}\sigma_{r^{y}}} \end{array}$$

Where cov(*r*^*x*^, *r*^*y*^) is the covariance of the rank variables and $\sigma_{r^{x}}$ and $\sigma_{r^{y}}$ are the standard deviations of the rank variables. For each pair of defensin and microbial species, the corresponding *p*-value was also calculated, which was further subject to multiple testing correction by the Benjamini and Hochberg method.

### Validating the diagnostic value of defensins on independent datasets

An independent cohort of 100 pneumonia patients (61 bacterial and 39 viral) were used to validate the diagnostic value of defensins and associated genes (NCBI Gene Expression Omnibus, access number: GSE40012) (Parnell et al., 2012). The whole blood gene expression profiles were quantified by Illumina HT-12 gene-expression beadarrays. Expression levels of defensins and associated genes were then extracted for clustering and classification analysis. For clustering analysis, t-distributed stochastic neighbor embedding (t-SNE) (van der Maaten and Hinton, 2008) was first used to reduce the dimensionality of the data to two for visualization and then a clustering method based on searching density peaks (Rodriguez and Laio, 2014) was used to cluster the samples into two groups. For classification analysis, the popular random forest method (Breiman, 2001) was used to evaluate the diagnostic value via a leave-one-out cross-validation method. The diagnostic value of defensins and associated genes was further validated on two additional independent datasets. One dataset included 12 children's admitted to *Streptococcus pneumoniae* or Staphylococcus aureus infections and 10 children's admitted to viral infections by influenza viruses (NCBI Gene Expression Omnibus, access number: GSE6269) (Ramilo et al., 2007). The other dataset included 67 bacterial and 113 viral infections for adults (NCBI Gene Expression Omnibus, access number: GSE63990) (Tsalik et al., 2016).

## Results

### Evident associations of different defensins to the bacterial and viral loads of H7N9 pneumonia patients

**Figure 1:**
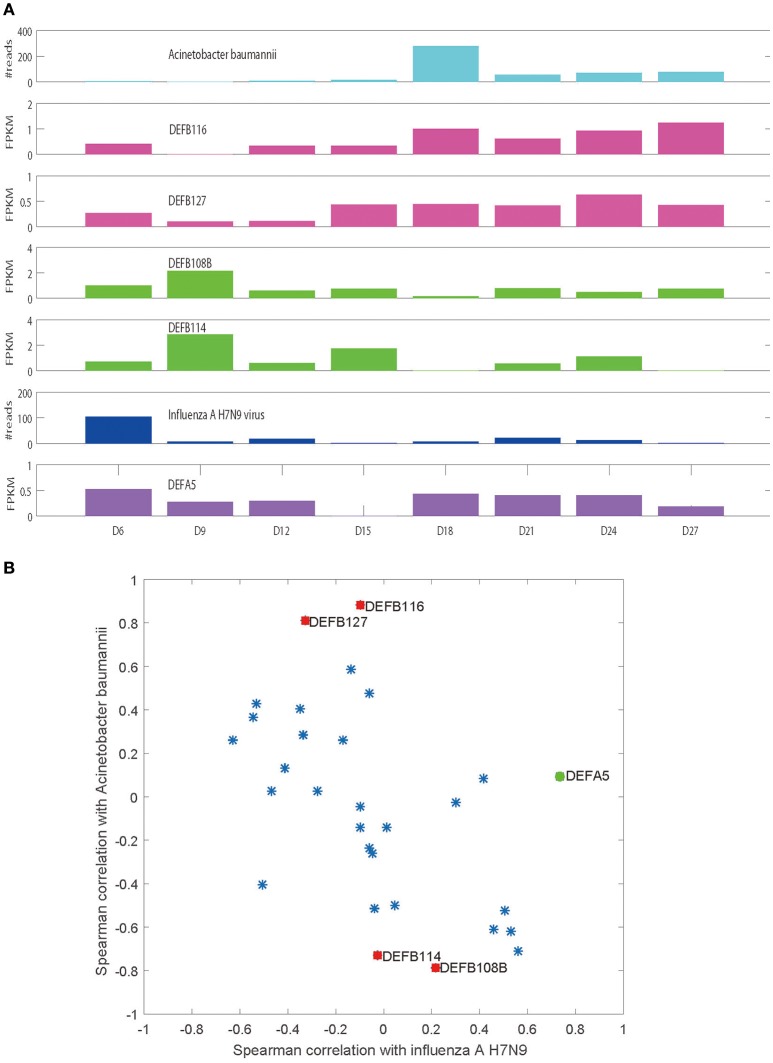
Spearman correlations of defensins and the viral/bacterial loads in blood. **(A)** Plots of the expression levels of selected defensins and the viral/bacterial loads along disease progression. **(B)** Spearman correlations of the total 30 defensins and the viral/bacterial loads.

It is evident that influenza H7N9 virus demonstrated two peaks in the patient blood (from Day 6 to Day 12 and from Day 18 to Day 24), with days from Day 12 to Day 18 forming a valley (Figure 1A). However, at Day 18, a huge peak of *Acinetobacter baumannii* infection appeared which declined in the following days with small fluctuations (Figure 1A). The total of 30 defensins measured (4 α and 26 β defensins) were all expressed in at least one sample or more (Table 1). Most of the defensins except DEFA5, DEFB116, DEFB127, DEFB114, and DEFB108B did not show correlations to or only showed weak correlations to viral/bacterial loads in blood that were statistically not significant (Figure 1B). DEFA5 was positively correlated to the blood load of influenza A H7N9 virus (*r* = 0.735, *p* < 0.05, Spearman correlation), which also showed two peaks similar to those of the virus (Figure 1A). But DEFA5 did not show correlations to the bacterial load. Different from DEFA5,

**Table 1:** The expression levels of the total 30 defensins and the viral/bacterial loads along disease progression.

	**Day6**	**Day9**	**Day12**	**Day15**	**Day18**	**Day21**	**Day24**	**Day27**
H7N9	105	6	17	1	6	22	13	1
Acinetobacter baumannii	3	0	7	12	279	54	69	76
DEFB4A	0.00	0.57	0.82	0.58	0.00	0.37	0.19	0.00
DEFB4B	0.00	0.00	0.00	0.00	0.00	0.25	0.00	0.00
DEFA4	0.00	0.08	0.26	0.57	0.08	0.15	0.08	0.52
DEFA3	0.14	0.11	0.24	0.00	0.00	0.00	0.11	0.00
DEFB131	1.32	3.14	1.13	1.08	0.00	2.04	2.06	0.00
DEFB1	0.46	0.48	0.39	0.25	0.00	0.47	0.47	0.48
DEFA5	0.52	0.28	0.30	0.00	0.44	0.40	0.41	0.18
DEFA6	0.47	0.37	0.67	0.38	0.00	0.12	0.49	0.49
DEFB136	0.90	2.14	0.00	0.73	2.26	0.70	0.00	0.94
DEFB135	2.82	2.24	0.00	0.77	2.36	0.73	0.73	1.97
DEFB133	2.24	0.00	0.00	1.83	0.00	0.00	0.00	2.35
DEFB116	0.40	0.00	0.34	0.32	0.99	0.61	0.92	1.24
DEFB115	0.00	0.48	0.00	0.99	0.00	0.94	1.42	1.91
DEFB114	0.72	2.86	0.62	1.77	0.00	0.56	1.12	0.00
DEFB113	0.00	0.00	1.30	0.62	0.00	1.18	1.19	0.00
DEFB112	0.00	0.24	0.52	1.74	1.27	0.00	0.24	0.00
DEFB110	0.70	0.18	2.20	0.76	0.20	0.36	1.82	2.45
DEFB134	4.95	0.61	4.39	2.10	0.48	0.33	0.34	1.58
DEFB121	0.68	1.24	1.16	0.45	0.60	0.88	0.71	0.34
DEFB132	0.56	0.54	0.51	0.42	0.45	0.35	0.50	0.67
DEFB128	0.75	0.00	0.00	0.41	1.04	0.58	0.00	0.78
DEFB125	0.32	0.26	0.46	0.44	0.27	0.59	0.59	1.02
DEFB123	0.63	0.13	0.41	1.16	0.66	0.37	0.37	0.83
DEFB119	2.59	1.47	0.58	0.82	0.88	0.52	1.34	2.32
DEFB124	1.93	1.80	3.41	1.56	0.66	0.96	4.44	0.00
DEFB108B	1.00	2.15	0.61	0.76	0.17	0.79	0.48	0.75
DEFB118	0.17	0.21	0.52	0.25	0.32	0.27	0.13	0.63
DEFB126	0.09	0.44	0.47	0.52	0.46	0.57	0.21	0.48
DEFB129	0.42	0.66	0.21	0.55	0.14	0.32	0.46	0.53
DEFB127	0.27	0.11	0.11	0.44	0.45	0.41	0.63	0.42

*Defensin expression levels were quantified by FPKM and the viral/bacterial loads were quantified by the number of reads*.

DEFB116 and DEFB127 were positively correlated to the blood load of *Acinetobacter baumannii* (*r* = 0.881 and 0.810, *p* < 0.05), both of which showed two peaks with one consistent with the peak of *Acinetobacter baumannii* and another at Day 6 (Figure 1A). The peak at Day 6 may indicate latent bacterial infection that was undetectable in blood, suggesting potentially superior sensitivity of defensin-based diagnostics. DEFB114 and DEFB108B showed negative correlations with *Acinetobacter baumannii* (*r* = −0.731 and −0.786, *p* < 0.05, Spearman correlation, Figures 1A,B).

### Diagnostic values of defensins on an independent pneumonia cohort

**Figure 2:**
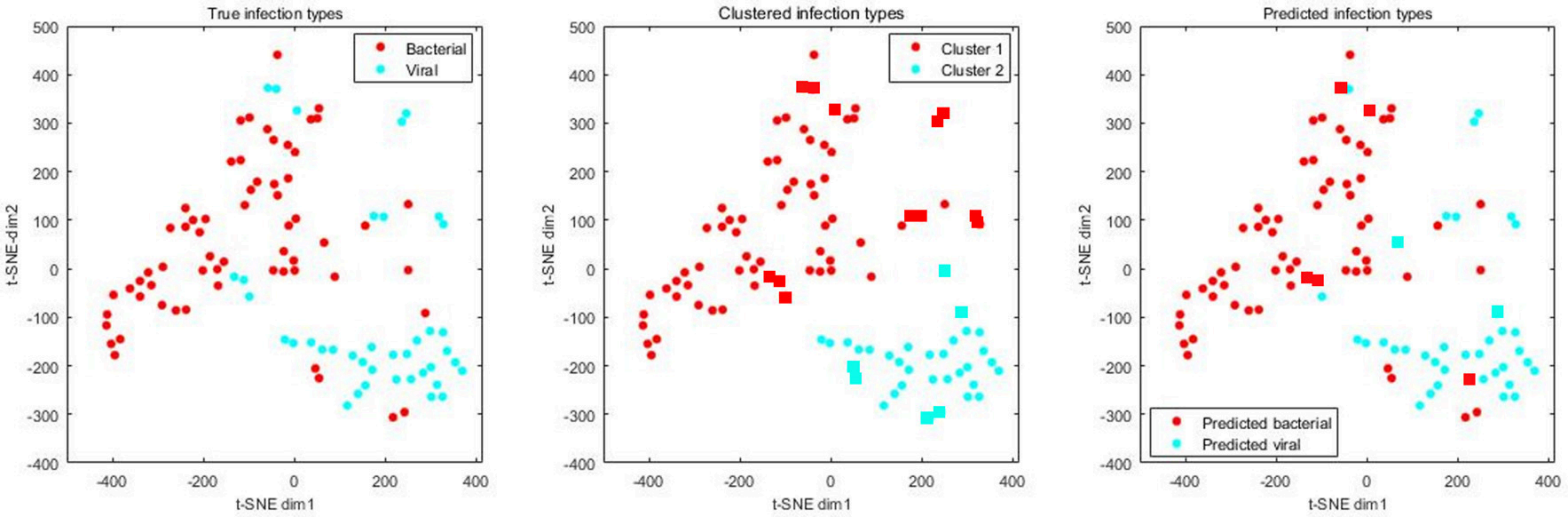
True, clustered and predicted infection types of 61 bacterial and 39 viral pneumonia patients. Expression levels of defensins and associated genes were extracted from the whole dataset and then subjected to t-SNE analysis for visualization. Circles mean correctly clustered/classified samples while rectangles mean incorrectly clustered/classified samples.

On the independent validation dataset, we first extracted the expression profiles of defensins and associated genes and conducted t-SNE for visualization. It is obvious that bacterial and viral pneumonia patients separately formed clusters with a few exceptions (Figure 2, left). Clustering analysis grouped the patients into two classes, one of which corresponded to bacterial pneumonia and the other corresponded to viral pneumonia (Figure 2, middle). The accuracy of clustering analysis reached 82%, with 18 patients mis-clustered. Clustering based on the raw high-dimensional data resulted in similar results, suggesting that bacterial and viral infections caused different responses for defensins and associated genes in blood. When switching the algorithms from unsupervised to supervised, high accuracy (93%), AUC (0.97), sensitivity (0.98), specificity (0.82), precision (0.90), and F1-score (0.94) were achieved by a random forest classifier with default parameters (Figure 2, right), suggesting the potential of defensin-based diagnostics to discriminate viral/bacterial infections.

**Figure 3:**
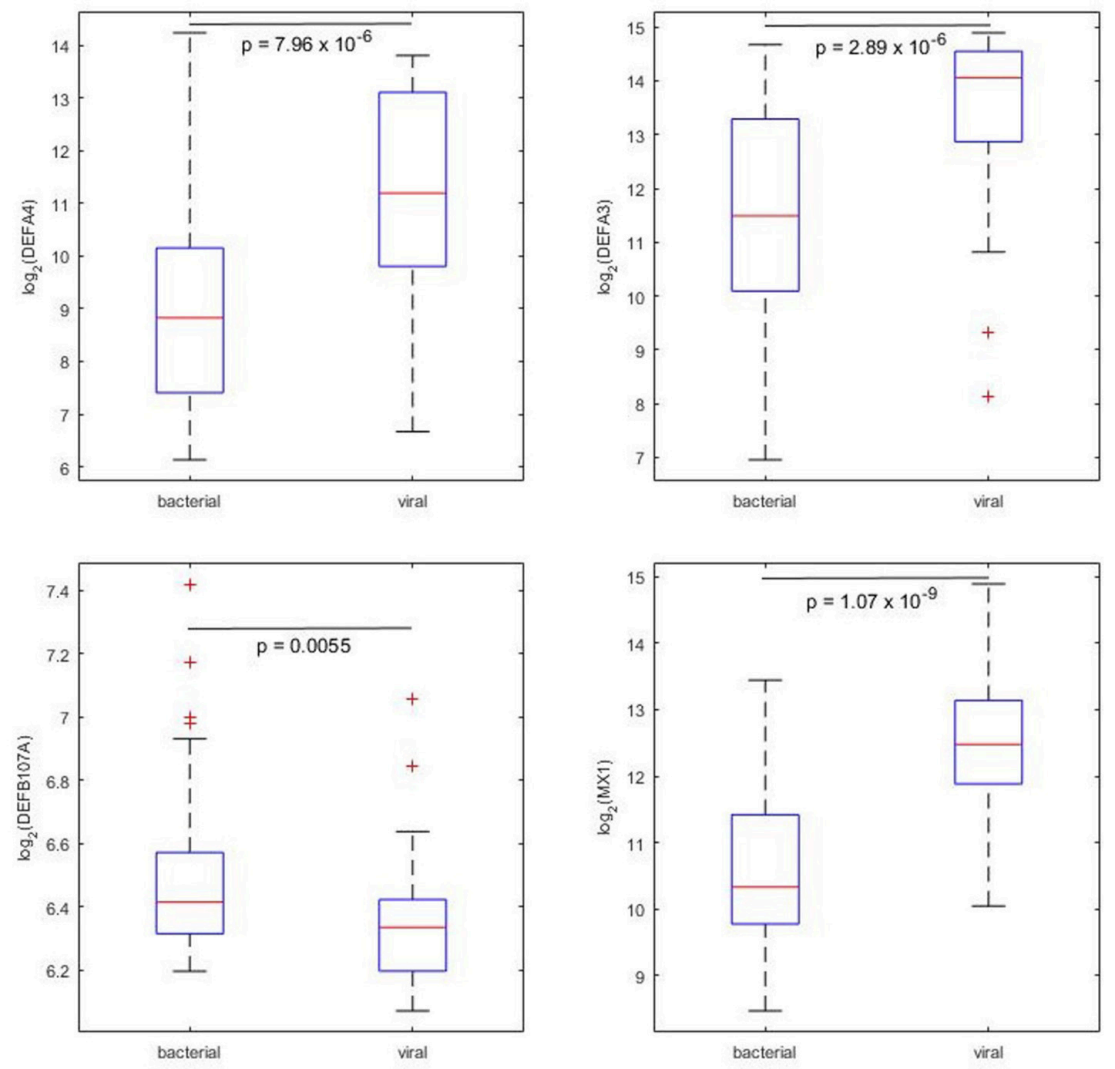
Four example defensins and associated genes that showed significant differences between bacterial and viral pneumonia patients (illustrated by boxplots).

Among the 87 defensins and associated genes that had expression values available, DEFA4 and DEFA3 were the most significantly differentially expressing defensins between bacterial and viral pneumonia patients. Both of these two defensins are alpha defensins and highly expressed in viral pneumonia patient blood (Figure 3, upper). The *p*-values tested by Wilcoxin rand-sum test were 7.96 × 10^−6^ and 2.89 × 10^−6^ for DEFA4 and DEFA3, respectively. DEFB107A was significantly highly expressed in bacterial pneumonia patient blood (Figure 3, lower left, *p* = 0.0055, Wilcoxin rand-sum test). MX1 is the most significant defensin-associated gene differentially expressed between bacterial and viral pneumonia (Figure 3, lower right, *p* = 1.07 × 10^−9^, Wilcoxin rand-sum test).

**Figure 4:**
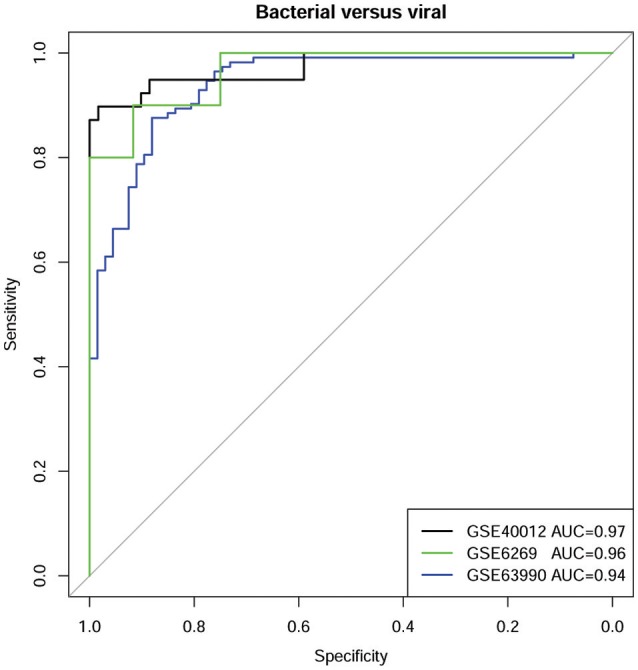
ROC curves of defensins and associated genes for classifying bacterial and viral infections on three datasets.

Evaluations on two additional datasets (GSE6269 and GSE63990) confirmed the diagnostic power of defensins and associated genes (Figure 4). On the dataset GSE6269, the accuracy can reach 95% while the AUC, sensitivity, specificity, precision, and F1-score are 0.96, 1, 0.9, 0.92, and 0.96, respectively. On the dataset GSE63990, similar performance was obtained, with accuracy 89%, AUC 0.94, sensitivity 0.84, specificity 0.93, precision 0.88 and F1-score 0.85.

## Discussion

Accurate discrimination of bacterial and viral infections has important clinical values and can inform clinicians to properly select therapies. Identifying biomarkers that can accurately classify bacterial infections from viral infections is thus of great importance. Blood-based assays including microarrays and next-generation sequencing provide a quite convenient method to quantify the expression levels of various genes, which form a rich resource for determination of biomarkers discriminating bacterial and viral infections. Multiple studies have been completed to seek such biomarkers from human blood gene expression profiles (Zaas et al., 2009; Parnell et al., 2012; Hu et al., 2013, 2015; Suarez et al., 2015). However, the values of defensins are often overlooked. Defensins, which are a major family of antimicrobial peptides expressed predominantly in neutrophils and epithelial cells and play important roles in innate immune defense against infectious pathogens, are hypothesized by us to action in distinct ways when combating against bacterial and viral infections, and thus we conducted this study.

We addressed the diagnostic values of defensins through two ways. Firstly, we checked the associations between human blood defensin mRNAs and the bacterial and viral loads through a continuous follow-up of a pneumonia patient caused by infection of influenza A H7N9 virus. This longitudinal study revealed that bacterial and viral loads were associated to beta and alpha defensins, respectively, among which several defensins showed impressing statistical significance. Secondly, we re-analyzed the diagnostic values of defensins on an independent dataset, which quantified blood gene expression profiles of 100 pneumonia patients including 61 bacterial and 39 viral infections. This lateral study demonstrated again the diagnostic power of defensins for discriminating bacterial and viral infections. Both studies remind that defensins and associated genes have great diagnostic potentials which deserve further investigation in the future. Although, the statistically significant defensins in these two studies did not overlap well, they could be caused or at least explained by the different study types and profiling techniques (microarray-based or NGS-based). Further studies were needed to exclude the technical interference and to include more biological variance.

We also compared the defensin-based biomarkers with published biomarker panels. We noticed that MX1 appeared multiple times across the studies, consistent with its great difference between bacterial and viral infections. Other defensins and associated genes are reported for the first time to have diagnostic power to discriminate bacterial from viral infections, and thus may provide new insights into the infection mechanisms and serve as important tools for clinical diagnosis. Because innate immunity is the first frontier of host to combat pathogens, the differences of defensins and associated genes during bacterial and viral infections may suggest that prominent patterns exist in host innate immune responses and defensins are valid representative molecules.

In summary, defensins not only are important molecules for hosts to combat infections, but also may provide promising biomarkers to indicate the types of infectious agents, which is expected to of significant clinical utility and needs further investigations.

## Author contributions

XR, JY, and QJ designed the experiment. SZ and XR performed the experiment. XR wrote the manuscript with all the authors contributing to the writing.

### Conflict of interest statement

The authors declare that the research was conducted in the absence of any commercial or financial relationships that could be construed as a potential conflict of interest.
